# Epigenetic Differentiation Persists after Male Gametogenesis in Natural Populations of the Perennial Herb *Helleborus foetidus* (Ranunculaceae)

**DOI:** 10.1371/journal.pone.0070730

**Published:** 2013-07-25

**Authors:** Carlos M. Herrera, Mónica Medrano, Pilar Bazaga

**Affiliations:** Estación Biológica de Doñana, Consejo Superior de Investigaciones Científicas, Sevilla, Spain; University of Leeds, United Kingdom

## Abstract

Despite the importance of assessing the stability of epigenetic variation in non-model organisms living in real-world scenarios, no studies have been conducted on the transgenerational persistence of epigenetic structure in wild plant populations. This gap in knowledge is hindering progress in the interpretation of natural epigenetic variation. By applying the methylation-sensitive amplified fragment length polymorphism (MSAP) technique to paired plant-pollen (i.e., sporophyte-male gametophyte) DNA samples, and then comparing methylation patterns and epigenetic population differentiation in sporophytes and their descendant gametophytes, we investigated transgenerational constancy of epigenetic structure in three populations of the perennial herb *Helleborus foetidus* (Ranunculaceae). Single-locus and multilocus analyses revealed extensive epigenetic differentiation between sporophyte populations. Locus-by-locus comparisons of methylation status in individual sporophytes and descendant gametophytes showed that ∼75% of epigenetic markers persisted unchanged through gametogenesis. In spite of some epigenetic reorganization taking place during gametogenesis, multilocus epigenetic differentiation between sporophyte populations was preserved in the subsequent gametophyte stage. In addition to illustrating the efficacy of applying the MSAP technique to paired plant-pollen DNA samples to investigate epigenetic gametic inheritance in wild plants, this paper suggests that epigenetic differentiation between adult plant populations of *H. foetidus* is likely to persist across generations.

## Introduction

Interest in the evolutionary and ecological significance of epigenetic processes has increased considerably in recent years [1]–[4]. In the case of plants, this upsurge of interest has been largely motivated by evidence that epigenetic markers often are not reset across generations [5]–[6], epigenetic differences can be an important source of heritable phenotypic variation [7]–[10], epigenetic variation can greatly exceed genetic differences between individuals or populations [11]–[16], and epigenetic processes can impinge on fitness-related traits and ecological processes at the individual, population and community levels [3], [17]–[19]. With few exceptions, however, inferences and speculations about the role of epigenetics in plant evolution have been based on investigations conducted in artificial environments using agricultural crop species or the model plant *Arabidopsis thaliana*. While the importance of such studies for understanding the mechanistic underpinnings of epigenetic processes cannot be overemphasized, insights from natural populations of non-model plants are also needed to evaluate the reality and possible consequences of epigenetic processes in the scenarios where populations live and possibly evolve [4], [20], [21].

The crux of the postulated evolutionary role of epigenetic variation lies in the assumption of gametic inheritance of epigenetically-based phenotypic variants [2], [6], [22], [23]. This key assumption has been often verified for cultivated and model plants under artificial conditions [10], [24]–[27], but practical difficulties have so far precluded extending similar tests to populations of non-model plants in their natural environments. The current lack of information on the transgenerational constancy of epigenetic characteristics of wild plant populations in natural scenarios has so far hindered interpretations of natural patterns of epigenetic variation in an evolutionary framework [20], [28]. This particularly applies to instances of epigenetic differentiation between populations of the same species living in contrasting environments [14], [15], [29], [30], whose evolutionary significance will critically depend on whether such potentially adaptive, epigenetic structuring persist across successive generations [4], [20], [31]. Evaluating transgenerational constancy of standing epigenetic structuring in wild plant populations, however, confronts some difficulties. Direct assessment through longitudinal sampling of successive generations of plants in the field will be in practice limited to short-lived species, and this approach will not allow to distinguish whether epigenetic similarity of offspring to parents actually reflects true gametic inheritance or the quick regaining of epigenetic features in response to shared environmental conditions. Common garden experiments similar to those used traditionally to evaluate the genetic basis of phenotypic population differences are not exempt from problems either. Given the alternation of diploid and haploid generations that characterizes the life cycle of higher plants, and the epigenetic responsiveness of germline to changes in the environment [23] such as those associated with transplants from natural to artificial environments, transplant experiments are prone to produce biased or distorted views of epigenetic inheritance.

We put forward in this paper an alternative procedure that allows a direct, genome-wide evaluation of transgenerational constancy in epigenetic population structure that can be directly applied to wild populations in their natural settings. The method consists basically of comparing patterns of DNA methylation and epigenetic population differentiation exhibited by adult plants and their pollen. In flowering plants the life cycle alternates between a diploid sporophytic phase and a haploid gametophytic phase, and germlines of individual sporophytes are not established until they approach reproductive maturity [32]. Epigenetic reprogramming, including changes in frequency and genomic distribution of cytosine methylation, may take place during gametogenesis, the process that connects the sporophytic and gametophytic generations [33]–[35]. A key requirement for gametic inheritance of a given epigenetic mark is therefore that the latter should go without change through the ‘epigenetic checkpoint’ set by gametogenesis [33]. Consequently, looking at the degree of epigenetic concordance between sporophytes and their descendant gametophytes may contribute useful insights on the transgenerational robustness of epigenetic features at the individual and population levels in natural environments, and on the extent and patterns of gametic inheritance of epigenetic characteristics as well. We present in this paper the results of applying this method to populations of the perennial herb *Helleborus foetidus* (Ranunculaceae) occurring along a natural ecological gradient in southeastern Spain. A comparative analysis of patterns of cytosine methylation in sporophyte (adult plants) and their descendant male gametophytes (pollen) was performed to evaluate the extent to which epigenetic reprogramming during gametogenesis obliterated epigenetic differentiation between adult plant populations. Our results revealed that epigenetic reorganization during the sporophyte-gametophyte transition, albeit substantial, was insufficient to erase epigenetic differences between adult plant populations.

## Materials and Methods

### Ethics statement

Field work for this study was conducted in publicly-owned, protected land of Parque Natural Sierras de Cazorla-Segura-Las Villas (Jaén province, southeastern Spain), under permits issued by Consejería de Medio Ambiente, Junta de Andalucía, Spain.

### Field methods

*Helleborus foetidus* L. (Ranunculaceae) is a perennial, evergreen herb widely distributed in western Europe, where it typically occurs in the understory of broad-leaved, coniferous and mixed forests [36], [37]. Plants consist of several ramets that develop terminal inflorescences asynchronously after 2–7 seasons of vegetative growth. In the area where this study was conducted (Sierra de Cazorla, Jaén province, southeastern Spain), the distribution of *H. foetidus* encompasses a broad range of elevations (700–1850 m a. s. l.) and vegetation types, from evergreen mediterranean forest at low elevations through deciduous broad-leaved forest at middle elevations to open pine woodland at highest locations. To enhance the likelihood of finding environmentally-driven epigenetic differentiation between populations, and assuming that changes in elevation should run parallel to changes in ecological conditions, we sampled three *H. foetidus* populations located at low (‘Tejerina’, TEJ hereafter; 730 m a. s. l., 37° 58.639′ N, 2° 54.424′W), middle (‘Las Navillas’, NAV hereafter; 1240 m a. s. l., 37° 56.156′ N, 2° 54.583′ W) and high (‘Puerto Llano’, PLL hereafter; 1800 m a. s. l., 37° 48.607′ N, 2° 57.557′ W) elevations along the species' altitudinal range. Horizontal distances between the nearest (TEJ-NAV) and farthest (TEJ-PLL) sampling sites were 4.6 km and 19.1 km, respectively.

At each site, 20 widely spaced, inflorescence-bearing plants were randomly selected for this study during February-May 2012. Sites differed significantly in average life history and reproductive traits of plants, including number of ramets, age of ramets at flowering, basal diameter of inflorescence axis, number of flowers produced per inflorescence, and individual flower size (Table S1). Elevational differences between sites were also associated with marked phenological differences. All locations were sampled at equivalent phenological stages, to avoid possible developmental variation in DNA methylation confounding individual or population differences in methylation patterns. Sampling was conducted at each site during the local flowering peak (February, March and May, for TEJ, NAV and PLL, respectively). All inflorescences on marked plants were bagged with fine mesh nets to prevent access to floral visitors that would remove pollen. Between 7–10 days later, when sufficient number of anthers had dehisced inside bagged flowers, paired leaf and pollen samples were collected from each plant. Young expanding leaves were cut, placed in paper envelopes and dried immediately at ambient temperature in sealed containers with abundant silica gel. Flowers of *H. foetidus* bear 35–45 stamens each producing ∼25000 pollen grains [38]. Pollen was collected by holding flowers tightly on top of microcentrifuge tubes that were vibrated manually. Pollen from different flowers on the same plant were pooled into a single sample. Pollen samples were carefully scrutinized immediately after collection to remove any remain of maternal tissue (e.g., anther walls), and dried at ambient temperature.

### Laboratory methods

Total genomic DNA was extracted from dry leaf (∼30 mg) and pollen (∼7 mg) material using Qiagen DNeasy Plant Mini Kit and the manufacturer protocol, with some minor modifications required for processing the small amount of material in pollen samples. Other extraction methods were assayed on pollen which resulted in better DNA yields (e.g., hotshot, lysis + protK, Qiagen DNeasy Blood & Tissue Kit), but we finally adopted the comparatively inefficient Plant Mini Kit method to avoid possible artifacts that could arise from extracting leaf and pollen DNA using different protocols. DNA concentration of extracts was estimated by running electrophoreses of 5 µL aliquots on 0.8% agarose gels. We used methylation-sensitive amplified polymorphism (MSAP) analysis to identify methylation-susceptible anonymous 5′–CCGG sequences and assess their methylation status. MSAP is a modification of the standard amplified fragment length polymorphism (AFLP) technique that uses the methylation-sensitive restriction enzymes *Hpa*II and *Msp*I in parallel runs in combination with another restriction enzyme (commonly *Eco*RI or *Mse*I; *Mse*I was used in this study because of its better repeatability in preliminary trials). *Hpa*II and *Msp*I are isoschizomers that recognize the same tetranucleotide 5′-CCGG but have differential sensitivity to methylation at the inner or outer cytosine. Differences in the products obtained with *Hpa*II and *Msp*I should thus reflect different methylation states at the cytosines of the CCGG sites recognized by *Hpa*II or *Msp*I cleavage sites (see [14], [15], [30], for recent applications of the MSAP technique to non-model plants).

We conducted MSAP assays on DNA samples from leaf and pollen material from the 60 *H. foetidus* individuals sampled. Standard AFLP analyses corresponding to each of the four *Mse*I + *Hpa*II and four *Mse*I + *Msp*I primer combinations assayed (Table 1) were performed as in [39]. Fragment separation and detection was made using an ABI PRISM 3130 xl DNA sequencer. The presence or absence of *Mse*I + *Hpa*II and *Mse*I + *Msp*I fragments in each sample was scored manually by visualizing electrophoregrams with GeneMapper 3.7 software. Only fragments ≥ 150 base pairs in size were considered to reduce the potential impact of size homoplasy [40]. Genotyping error rates were computed separately for each fragment by running repeated analyses for 36 samples (30% of total), and estimated as the ratio of the number of discordances to the number of samples scored. Only fragments with error rates lower than the median of the error distribution for the whole set of fragments were retained for further analysis (*N* = 143), and mean genotyping error rates were then determined separately for each primer combination.

**Table 1:** Primer combinations used, number of AFLP loci in the size range 150–500 bp, scoring error rates, and frequency and polymorphism of non-methylated and methylation-susceptible loci, in the methylation-sensitive amplified polymorphism (MSAP) analysis of 60 adult plants of *Helleborus foetidus* from three populations considered in this study.

Primer combination	Number of AFLP loci	Scoring error rate (%) 1	Non-methylated loci		Methylation-susceptible loci		
			N	% polymorphic	N	% polymorphic	
C1. *Mse*I + CGT/*Hpa*II-*Msp*I + TT	36	4.1	14	7.1	22	40.9	
C2. *Mse*I + CTT/*Hpa*II-*Msp*I + TC	44	4.2	7	71.4	37	54.1	
C3. *Mse*I + CGT/*Hpa*II-*Msp*I + TA	47	4.6	11	9.1	36	36.1	
C4. *Mse*I + CGA/*Hpa*II-*Msp*I + TG	16	5.2	4	25.0	12	25.0	
All combined	143	4.4	36	22.2	107	42.1	

1 Based on data for 36 samples that were assayed twice, computed as 100× (number of discordant scores on two independent analyses)/(number of scored markers x number of samples).

### Data analysis

MSAP analyses followed closely the methods described in detail by [14], as implemented in the msap package [41] for the R environment [42]. In a first step, element-wise comparisons were performed of fragment presence-absence matrices obtained with *Mse*I-*Hpa*II and *Mse*I-*Msp*I primer combinations. Each fragment was then classed as either “methylation-susceptible” or “non-methylated”, depending on whether the proportion of discordant scores obtained with *Hpa*II and *Msp*I exceeded the estimated threshold of obtaining a *Hpa*II–*Msp*I mismatch due solely to scoring errors (i.e., drawing a false inference of methylation). Only data for methylation-susceptible fragments were considered in the rest of analyses. The methylation status of every fragment in each sample was determined depending on whether the fragment was (i) present in both *Mse*I-*Hpa*II and *Mse*I-*Msp*I products; (ii) absent from both *Mse*I-*Hpa*II and *Mse*I-*Msp*I products; or (iii) present only in either *Mse*I-*Hpa*II or *Mse*I-*Msp*I products. Condition (i) denotes a non-methylated state, condition (iii) corresponds to a methylated state (hemimethylated or internal cytosine methylation), and condition (ii) is uninformative, since it could be due to either fragment absence or hypermethylation [43], [44]. The DNA-methylation fingerprint was obtained for each sample by scoring fragments as if the methylated state were an imperfectly assessed dominant marker: 1, for the methylated state (condition iii above); 0, for the non-methylated state (condition i above), and unknown (i.e., score missing) for uninformative condition ii above.

Single-locus and multilocus analyses of epigenetic differentiation of *H. foetidus* between sampling sites were tested, respectively, using Fisher exact probability tests for population heterogeneity in methylation frequency and analyses of molecular variance (AMOVA) [45]. Given the multiplicity of simultaneous tests involved in single-locus analyses, the *q*-value method was used to estimate false discovery rates. Using the qvalue package for R [46], we calculated the *q*-values for individual tests, and considered statistically significant only those with *P*-value and *q*-value simultaneously <0.05. AMOVA computations were conducted using the amova function in the pegas package for R [47]. Analyses were based on pairwise distance matrices whose elements were the squared distances between the corresponding vectors of binary scores, obtained using dist function in the stats package with method set to ‘euclidean’ to obtain metric distance matrices as required by AMOVA [48]. Φ_st_ values, an analogue of *F*_st_ likewise measuring genetic differentiation, were also obtained from AMOVA analyses, and significance determined by random permutations [45]. Nonmetric multidimensional scaling [49] of epigenetic distance matrices, computed using the isoMDS function in the R package MASS [50], was used to obtain simplified multilocus representations of sporophyte and gametophyte samples on two-dimensional spaces. Because nonmetric multidimensional scaling prioritizes preservation of ordering relationships between objects, it is better than other distance-based ordination methods (e.g., principal coordinate analysis) at compressing the distance relationships among objects into low-dimensionality spaces [49].

Significance of the effects of locus and sampling site on the probability of occurrence of a methylation change between sporophyte and descendant gametophyte was tested by fitting a generalized linear model to methylation change data. Computations were performed using SAS procedure GLIMMIX, with binomial distribution for errors, logits as link function, residual pseudolikelihood estimation, and the default containment method for computing denominator degrees of freedom [51]. The assumption of locus independence implicit in this analysis was deemed reasonable in view of the frequent finding of AFLP markers being fairly uniformly, independently distributed across plant genomes [52], [53].

Instability of methylation status was estimated for each methylation-susceptible locus as the proportion of individual plants (all sites combined) that exhibited a methylation change for that locus between the sporophyte and descendant pollen gametophyte stages. The null hypothesis that the distribution of these per-locus instability values (arcsin transformed for normalization) had a single mode against the alternative hypothesis that it had ≥2 modes was tested using Hartigans' dip test [54], as implemented in R package diptest. Significant departure from unimodality of the per-locus instability distribution would reveal the existence of a mixture of distinct groups, or ‘populations’, of loci with different modal instability. After rejecting unimodality of the distribution, we classified loci into either ‘stable’ (low modal instability) or ‘unstable’ (high modal instability) clusters using normal mixture modeling via EM, model-based clustering with R package mclust [55].

## Results

### Frequency of methylation-susceptible loci

A total of 143 AFLP fragments (“loci” hereafter) could be reliably scored in DNA samples from leaves of the 60 *H. foetidus* individuals studied (Table 1). Proportion of *Hpa*II–*Msp*I discordances was lower or equal than the corresponding combination-specific error threshold for 36 loci (non-methylated loci, 25.2% of total), and exceeded the threshold for 107 loci (methylation-susceptible loci hereafter, 74.8% of total). Proportions of non-methylated and methylation-susceptible loci did not vary significantly among primer combinations (*P* = 0.14, Fisher exact probability test). Only the 107 methylation-susceptible (‘MS’ hereafter) loci will be considered in the rest of analyses.

### Epigenetic variation: sporophytes

There was extensive epigenetic polymorphism in *H. foetidus* sporophyte populations (‘plants’ hereafter), with 42.1% of the 107 MS loci being polymorphic (Table 1). Both single-locus and multilocus analyses revealed extensive epigenetic differentiation between sites. When analyzed individually, 24 polymorphic MS loci (53.3% of total) exhibited statistically significant variation among sites in proportions of methylated and non-methylated states (*P*≤0.039 and *q*-value ≤0.0024 in all cases; expected number of false positives = 0.0024×24 = 0.058).

**Figure 1:**
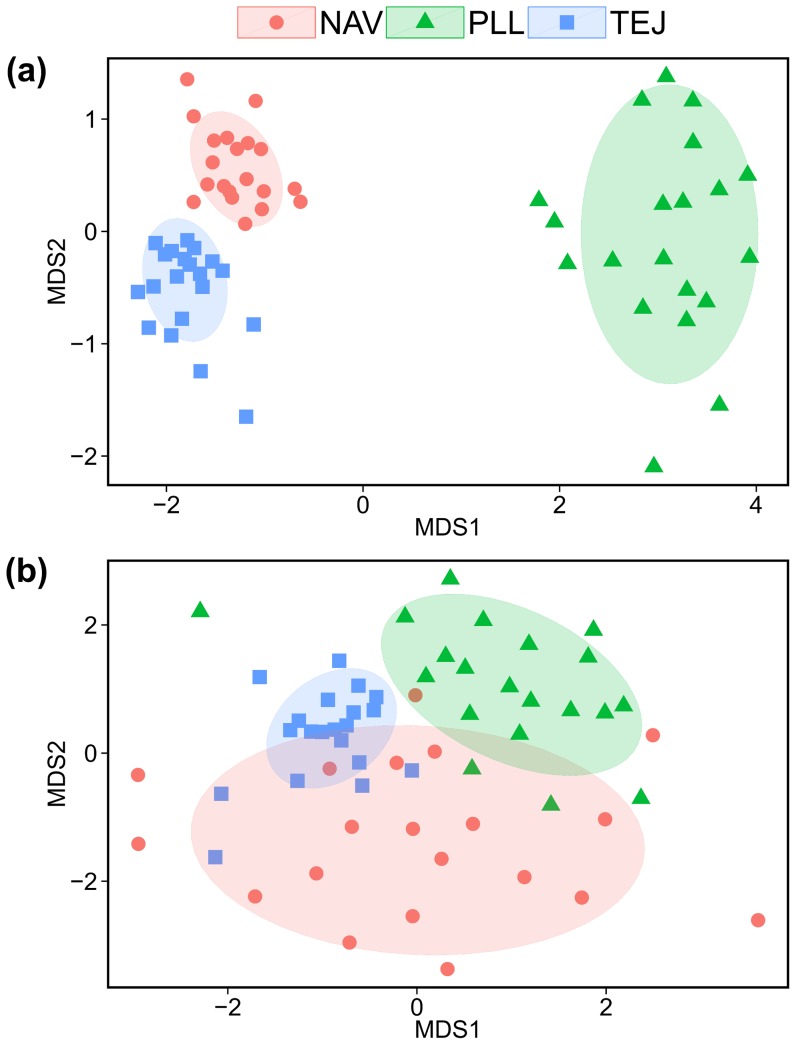
Epigenetic structure of sporophyte and descendant gametophyte populations. Scatterplot of *Helleborus foetidus* sporophyte (**a**) and descendant male gametophyte (**b**) populations on the plane defined by nonmetric multidimensional scaling axes (MDS1 and MDS2) from the respective pairwise matrices of epigenetic distance. Each symbol represents a sample, coded according to sampling site (NAV, TEJ and PLL), and ellipses denote the 95% bivariate confidence intervals around site means.

As expected from the high level of single-locus epigenetic differentiation, multilocus plant epigenotypes varied considerably between sites, as illustrated by the non-overlapping distribution of samples on the plane defined by the first two axes from the nonmetric multidimensional scaling analysis of the epigenetic distance matrix (Fig. 1a). Multilocus epigenetic heterogeneity between sites was highly significant (Φ_ST_ from AMOVA = 0.286, *P*<0.0001), with about 28% of total epigenetic variance in the sample being accounted for by between-site differences. The three Φ_ST_ values for pairwise comparisons between sites were also statistically significant (range of Φ_ST_ = 0.075–0.382; *P*<0.0001 in all cases).

### Epigenetic variation: descendant male gametophytes

There were both single-locus and multilocus epigenetic differentiation across sites of the male gametophyte populations (‘pollen’ hereafter) produced by the 60 adult plants included in this study. Twenty polymorphic MS loci (44.4% of total) exhibited statistically significant differences among sites in proportions of methylated and non-methylated states (*P*≤0.049 and *q*-value≤0.0012 in all cases; expected number of false positives = 0.0012×20 = 0.024). Multilocus epigenotypes of pollen populations from the three sites occupied distinct, albeit slightly overlapping regions in the space defined by nonmetric multidimensional scaling of the pairwise epigenetic distance matrix (Fig. 1b). Between-site multilocus epigenetic heterogeneity of pollen was highly significant (Φ_ST_ from AMOVA = 0.196, *P*<0.0001), about 20% of total epigenetic variance being accounted for by between-site differences. The three Φ_ST_ values for pairwise comparisons between sites were also statistically significant (range of Φ_ST_ = 0.169–0.255; *P*<0.0001 in all cases).

### Sporophyte-to-gametophyte changes: single-locus patterns

The methylation status of a given locus in leaves and pollen from the same individual could be compared in 5071 instances (all individuals and MS loci combined). This figure is smaller than the maximum possible (6420 = 60 plants×107 MS loci) because comparisons involving simultaneous occurrence of the uninformative condition in plant and pollen were discarded (i.e. fragment absence from both *Mse*–*Hpa* and *Mse*–*Msp* products). When only one uninformative score was involved in a comparison, presence of the fragment in leaf or pollen was used to solve ambiguity for pollen or leaf, respectively.

**Figure 2:**
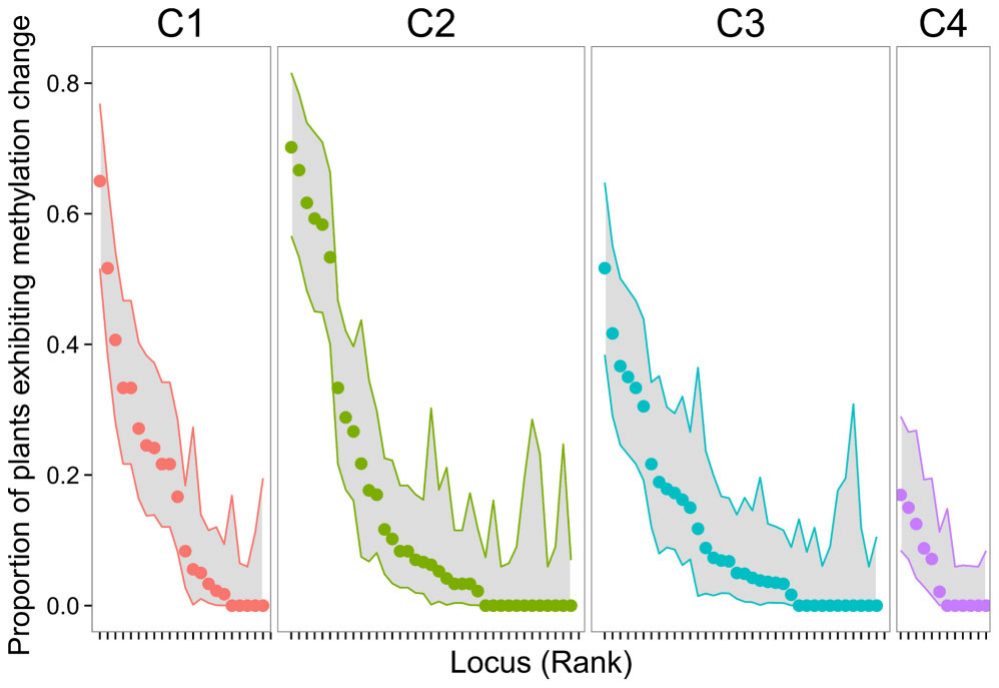
Frequency of DNA methylation changes over the sporophyte-gametophyte transition. Variation among methylation-susceptible loci in degree of instability of methylation status from sporophytes to descendant pollen gametophytes in *Helleborus foetidus*, expressed as the proportion of individual plants (all sampling sites combined) that exhibited a sporophyte-to-gametophyte methylation change for a given locus. Loci from the four primer combinations used (C1–C4, Table 1) are plotted separately to emphasize the shared pattern. Shaded areas represent the 95% binomial confidence intervals around estimates. Due to missing comparisons (both sporophyte and descendant pollen gametophyte exhibiting uninformative methylation scores), loci differed in number of plants entering the computations, thus the irregularities in width of confidence intervals.

Regardless of primer combination, most MS loci had their methylation status unchanged over the sporophyte-gametophyte transition in most individuals, with only a small subset of loci exhibiting high instability in many individuals (Fig. 2). Only 16.0% of all sporophyte-gametophyte comparisons (all plants and loci combined, *N* = 5071 comparisons) involved a change in methylation status. In most instances where a change took place, it implicated a transition from non-methylated to methylated status (80.3% of changes), the reverse transition occurring considerably less often (19.7%). The null hypothesis that the distribution of per-locus instability values (proportion of plants exhibiting a methylation change from sporophyte to pollen gametophytes for a given locus) had a single mode was rejected in favor of the alternative hypothesis that it had ≥2 modes (D = 0.0677, *P* = 0.00068; Hartigans' dip test). A normal mixture model fitted to per-locus instability data classed 80 (74.8% of total) and 27 (25.2%) MS loci into ‘stable’ (low modal instability) and ‘unstable’ (high modal instability) groups, respectively. Roughly three quarters of individual MS loci thus tended to predominantly retain their methylation status past male gametogenesis.

When modeled as a binomial process, the estimated probability of a single sporophyte- gametophyte methylation change (i.e., involving one locus in one individual) differed significantly between sites (*F*_2,4962_ = 7.80, *P* = 0.0004) and loci (*F*_72,4962_ = 8.08, *P*<0.0001). The model-estimated, mean per-locus probability of sporophyte-gametophyte methylation change (± SE) increased significantly from TEJ (0.00064 ± 0.00011) through PLL (0.00076 ± 0.00014) to NAV (0.00098 ± 0.00017). Sites differed also in the mean proportion of loci per individual that experienced sporophyte-gametophyte changes, which increased from TEJ (0.137 ± 0.006) through PLL (0.162 ± 0.008) to NAV (0.180 ± 0.009) (χ^2^ = 13.49, df = 2, *P* = 0.0012; Kruskal-Wallis rank sum test).

### Sporophyte-to-gametophyte changes: multilocus patterns

**Figure 3:**
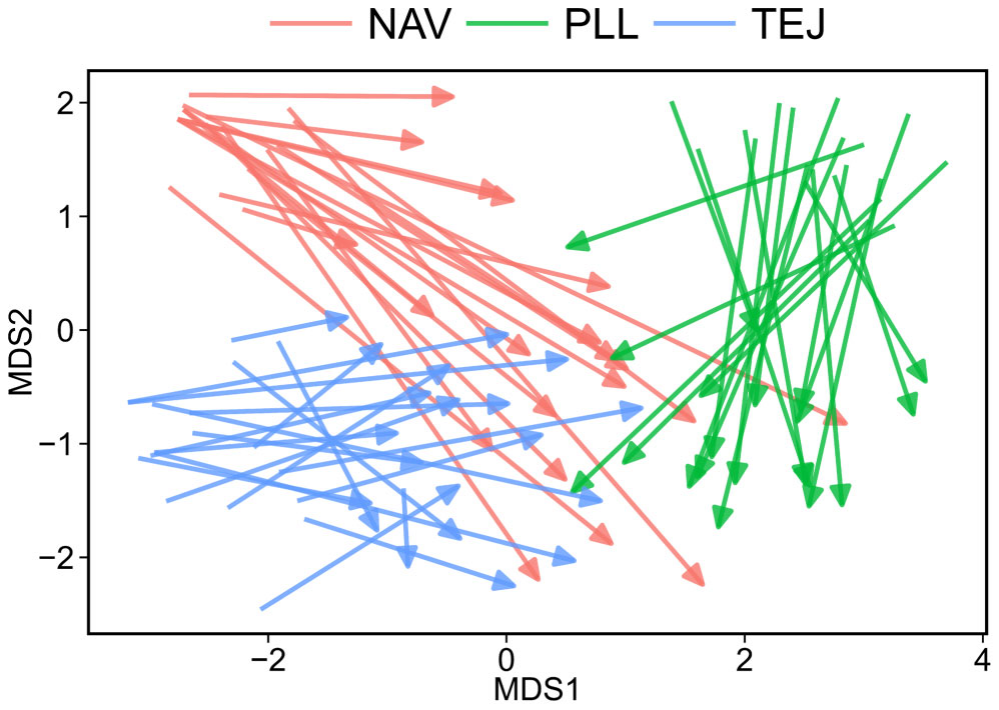
Epigenetic changes from individual sporophytes to descendant gametophytes. Scatterplot of *Helleborus foetidus* sporophyte and gametophyte samples on the common multilocus space defined by nonmetric multidimensional scaling axes (MDS1 and MDS2) from the pairwise epigenetic distance matrix for sporophytes and gametophytes combined into a single sample. Only data for the *N* = 29 loci whose methylation status differed significantly between sites for either sporophytes (*N* = 24) or gametophytes (*N* = 20) in single-locus analyses were included in the analysis. Each arrow connects the positions of the sporophyte (base) and its descendant gametophyte (tip), color-coded according to sampling site (NAV, TEJ and PLL). Note that this plot is not strictly comparable to the two plots in Fig. 1, as the latter are based on separate, independent analyses for sporophyte and gametophyte samples using the whole set of markers.

Methylation changes taking place from sporophytes to descendant gametophytes caused some reorganization of multilocus epigenetic differences between sites. This is clearly seen by plotting plant and pollen samples on a common multilocus space obtained from the nonmetric multidimensional scaling analysis of the pairwise distance matrix for sporophyte and gametophyte data combined (Fig. 3). To better highlight the alteration of between-site differences that take place from the sporophyte to the gametophyte stage, only data from the 29 loci whose methylation status differed across sites in plants and/or pollen were used to compute the common sporophyte-gametophyte multilocus space. Differences in length and direction of arrows connecting sporophyte and gametophyte samples for the same individual (Fig. 3) reveal extensive variation in pattern and magnitude of transgenerational, multilocus epigenetic reorganization. The combined result was reduced multilocus population differentiation, closer clustering of samples, and multilocus ‘convergence’ of gametophytes in relation to sporophytes (Fig. 3).

The trend towards a post-gametogenesis epigenetic convergence of all sites did not completely obliterate, however, the initial multilocus differentiation between sporophyte populations, as denoted by the following results based again on the 29 loci whose methylation status differed significantly between sites for plants and/or pollen. First, as many as 15 loci with methylation status varying significantly between sites did so for both sporophytes and gametophytes. Second, multilocus epigenetic heterogeneity of gametophytes between sites was highly significant (Φ_ST_ from AMOVA = 0.207, *P*<0.0001). And third, a highly significant correlation existed between the pairwise epigenetic distance matrices between individuals obtained separately for sporophytes and gametophytes (*r* = 0.288; *P*<0.0001, Mantel test with 10000 permutations). Taken together, these results highlight the transgenerational stability of epigenetic differentiation between *H. foetidus* populations in the face of the substantial reorganization taking place during gametogenesis.

## Discussion

Epigenetic characteristics of *H. foetidus* plants sampled for this study varied significantly across sampling sites, as found also by the few investigations that have so far looked for structuring of epigenetic variation in wild plant populations [14], [15], [29], [30]. Both single-locus and multilocus analyses revealed extensive differentiation between sites in the epigenetic characteristics of *H. foetidus* plants, with roughly 28% of total epigenetic variance in the sample being accounted for by differences between sites. This level of epigenetic differentiation is somewhat higher than other values reported for wild plants (13% and 21% for *Viola cazorlensis* and *Alternanthera philoxeroides*, respectively; [14], [30]), a result that probably reflects our non-random selection of sampling sites, which were chosen to maximize the breadth of ecological conditions faced by plants. Given the well-known effects of environmental stresses on patterns and extent of DNA methylation [56], [57], the transgenerational stability of epigenetic effects of stress [58], and the stressful conditions commonly faced at margins of altitudinal ranges [59], then the inclusion in our sampling of the uppermost and lowermost distributional margins of *H. foetidus* most likely contributed to enhance epigenetic differentiation between populations. On the other hand, the extensive epigenetic differentiation exhibited by the three *H. foetidus* populations studied here agrees with the recent hypothesis positing a functional link between epigenetic diversity and ecological breadth in wild plant populations [17]. Properly testing such epigenetic-ecological correlation for *H. foetidus* in our study region, however, will require a more thorough sampling of individuals and populations encompassing the whole ecological range of the species.

In contrast to animals, where reproductive and somatic cell lineages diverge early in embryogenesis and gametes are produced directly from diploid germlines, higher plants lack a differentiated germline, and reproductive and vegetative structures share a common cell lineage until late developmental stages [32], [60]. In close parallel with its long-acknowledged role in facilitating the transmission of somatic cell mutations to gametes [61], [62], late developmental appearance of germline in plants will also facilitate the transmission of epigenetic marks in nuclear DNA from somatic cells to gametes. Apart from its potential evolutionary significance [6], [23], [31], this circumstance opens practical opportunities for epigenetic research on natural plant populations, as illustrated by this study. Mature pollen grains are either bi- or trinucleate (binucleate in *H. foetidus*; [63]), containing one vegetative cell and one or two sperm cells. Methylation differences may arise between nuclear types, involving DNA demethylation in the vegetative nuclei that do not provide DNA to the fertilized zygote while sperm cells are not demethylated [34], [64], [65]. These differences would most likely add some ‘noise’ to the MSAP results for pollen, and lead to underestimation of marker transmission from sporophyte to gametophyte by the method used here. No information is available on possible methylation differences between the two nuclei in *H. foetidus* pollen grains, but even if such differences existed their influence on results was probably negligible in view of the high proportion of constant markers found. This leads us to conclude that, since DNA methylation in germ cells do not seem to experience major reprogramming after meiosis [33], [34], [66], the application of MSAP to pollen DNA is a useful technique to investigate patterns of gametic inheritance of natural epigenetic variation in plants that, like *H. foetidus*, have limited genetic and genomic resources.

Multilocus epigenetic differentiation exhibited by adult plant populations [14], [15] can be envisaged as the aggregate outcome of population differences in the methylation status of both gametically heritable and non-heritable (i.e., reset after meiosis) loci. Under this admittedly simplified framework, transgenerational robustness of epigenetic differentiation between populations will be directly related to the proportion of loci whose methylation status remain unchanged through the meiosis and postmeiotic mitoses that lead to gametophytes. Locus-by-locus comparisons of methylation status in individual sporophytes and its descendant gametophytes allowed us to estimate that, in wild-growing *H. foetidus* plants, a majority (∼75%) of anonymous epigenetic markers generally escaped modification of their methylation status in the transit between generations, a figure comparable to that obtained for other plants under artificial growing conditions [26], [33], [66], [67]. The predominant robustness through gametogenesis of single-locus methylation status ultimately accounted for our finding that, although a certain amount of epigenetic reprogramming during gametogenesis blurred population differentiation, the significant multilocus epigenetic differentiation between *H. foetidus* populations was still preserved from the sporophyte to the gametophyte stage.

The preservation in the male gametophyte stage of multilocus epigenetic differentiation between sporophyte populations leads us to predict that epigenetic differentiation exhibited by adult plant populations of *H. foetidus* studied here should still be discernible in the next few generations of sporophytes. This expectation assumes that (i) epigenetic population differentiation is preserved as well through female gametogenesis, and (ii) male-contributed DNA methylation patterns are not substantially supressed or counteracted in the diploid embryo by female-contributed ones through extensive maternal imprinting effects [68]. We were unable to test directly the first assumption for *H. foetidus* because individual ovules were so tightly associated with surrounding maternal tissue (funicle) that it proved impractical to obtain DNA samples exclusively from the female gametophytes. In *Arabidopsis*, however, DNA methylation changes during female gametogenesis largely affect the two central cell nuclei from which the endosperm develops, rather than the egg cell that will participate in embryo formation [34], [35], thus supporting our first assumption. The possibility of pervasive maternal imprinting in *H. foetidus* embryos suppressing or counteracting male-contributed DNA methylation patterns seems unlikely judging from available information for *Arabidopsis*, where maternal-only imprinting is almost exclusively associated with the endosperm and very few imprinted genes occur in the embryo [33], [34].

One revealing result of our study on *H. foetidus* is that, rather than being a deterministic, all-or-nothing process, the transmission from sporophyte to gametophyte of the methylation status of individual loci had a distinct stochastic component. Stochasticity occurred at various levels. The distribution of the per-locus probability of methylation change was significantly bimodal, which denoted the existence of two stability classes of loci (i.e., predominantly stable and predominantly unstable), yet some loci had intermediate modification rates and did not fall clearly into these classes (Fig. 2). Furthermore, mean genome-wide probability of sporophyte-to-gametophyte change in methylation status of individual loci differed significantly between sampling sites, and degree of transgenerational stability of epigenotypes at the multilocus level also differed between individuals, as clearly denoted by the broad variation in the length of arrows depicted in Fig. 3. Laboratory experiments have often revealed a certain degree of stochasticity in the sporophyte-to-gametophyte transmission of the methylation status of individual cytosines, whereby DNA methylation patterns do not fluctuate randomly from one generation to the next but neither are they completely stable [28], [66], [69]. Laboratory investigations on plant epigenetics, however, have often tended to convey a deterministic view of transgenerational transmission of individual epigenetic marks, possibly a consequence of these studies generally considering only one or a few genotypes to highlight purely epigenetic effects independent of genetic variation [10], [26], [28], [70]. In contrast, the large number of genotypes considered in this study have most likely facilitated the discovery of individual and population differences in the transmission probability of a significant number of epigenetic marks, thus highlighting the importance of studying epigenetic variation with a population perspective [20], [21]. More importantly, since epigenetic inheritance during male gametogenesis is under both genetic and epigenetic control [32], [35], [71], our finding that individuals and populations of *H. foetidus* differ in the ability to pass epigenetic marks unchanged through gametogenesis suggests the hypothesis that such ability may itself become a target for natural selection. This would particularly apply in ecological situations where epigenetic marks acquired sometime during the sporophyte life stage in response to biotic or abiotic stress would enhance parental fitness if passed unchanged to the germline. Under such ecological circumstances, and assuming that one genotype's capacity for transgenerational epigenetic inheritance is itself heritable, natural selection could drive adaptive epigenetic changes, which would eventually lead to epigenetic differentiation being linked to adaptive genetic divergence, a pattern that has been documented for the violet *Viola cazorlensis* [14]. In the case of *H. foetidus* populations in our study region, a first step towards assessing the merits of this hypothesis should include genome-wide association analyses to identify the genetic and epigenetic correlates of population and individual differences in ability to transmit epigenetic marks unchanged to descendant gametophytes, as well as their possible correlations with phenotypic differences between populations.

## Supporting Information

Table S1 Vegetative and reproductive characteristics of *Helleborus foetidus* plants sampled for this study.
(DOC)
