# Supplementary material for: Epigenetic Differentiation Persists after Male Gametogenesis in Natural Populations of the Perennial Herb Helleborus foetidus (Ranunculaceae)
Source: PLoS One. 2013 Jul 25;8(7):e70730. doi: 10.1371/journal.pone.0070730 (PMC3723889; doi:10.1371/journal.pone.0070730)
Supplement: Table S1 — Vegetative and reproductive characteristics of Helleborus foetidus plants sampled for this study. (DOC) [file pone.0070730.s001.doc]

**Table S1.** Vegetative and reproductive characteristics of adult *Helleborus foetidus* plants sampled for this study. *N* = 20 plants sampled per site. Diffences between sites were tested using Wilcoxon rank-sum tests.

|  | Site (elevation) | | |  | Difference | |
| --- | --- | --- | --- | --- | --- | --- |
|  | TEJ (730 m) | NAV (1240 m) | PLL (1800 m) |  | χ^2^  | *P* |
| Number of ramets per plant | 4.6 ± 0.4 | 6.2 ± 0.6 | 11.1 ± 1.2 |  | 23.62 | < 0.0001 |
| Age of flowering ramets (years) | 3.2 ± 0.2 | 5.0 ± 0.2 | 3.6 ± 0.2 |  | 27.27 | < 0.0001 |
| Basal diameter of inflorescence (mm) | 12.9 ± 0.6 | 10.8 ± 0.4 | 12.7 ± 0.4 |  | 10.24 | 0.006 |
| Flowers per inflorescence | 40.0 ± 3.2 | 30.2 ± 1.8 | 30.5 ± 2.3 |  | 5.48 | 0.065 |
| Flower length (mm) | 15.7 ± 0.2 | 17.0 ± 0.1 | 16.9 ± 0.1 |  | 20.95 | < 0.0001 |
